# Long-Read Nanopore Sequencing Identifies Mismatch Repair-Deficient Related Genes with Alternative Splicing in Colorectal Cancer

**DOI:** 10.1155/2022/4433270

**Published:** 2022-07-21

**Authors:** Hao Qu, Zhenjun Wang, Yudong Zhang, Baocheng Zhao, Shuai Jing, Jianwei Zhang, Chunxiang Ye, Yaohan Xue, Lei Yang

**Affiliations:** ^1^Department of General Surgery, Beijing Chao-Yang Hospital, Capital Medical University, Beijing 100020, China; ^2^Department of Colorectal and Anal Surgery, Beijing Rectum Hospital, Beijing 100120, China; ^3^Medical Research Center, Beijing Chao-Yang Hospital, Capital Medical University, Beijing 100020, China

## Abstract

**Background:**

Alternative splicing (AS) plays a crucial role in regulating the progression of colorectal cancer (CRC), but its distribution remains to be explored. Here, we aim to investigate the genes edited by AS which show differential expression in patients with mismatch repair deficiency (dMMR)/microsatellite instability (MSI).

**Materials and Methods:**

We applied long-read nanopore sequencing to determine the mRNA profiles and screen AS genes using Oxford Nanopore Technologies (ONT) method in ten paired CRC tissues. CRC tissue and plasma samples were used to validate the differential genes with AS using real-time fluorescent quantitative PCR, immunohistochemistry, and enzyme-linked immunosorbent assay.

**Results:**

ONT sequencing identified 404 genes were downregulated, and 348 genes were upregulated in MSI cancer tissues compared with microsatellite stability (MSS) cancer tissues. In total, 6,200 AS events were identified in 2,728 mRNA transcripts. WGCNA revealed dMMR/MSI-correlated gene modules, including INHBA and RPL22L1, which were upregulated; conversely, HMGCS2 was downregulated in MSI cancer. Overexpression of RPL22L1, INHBA, and CAPZA1 was further confirmed in CRC tissues. INHBA was found to be associated with tumor lymphatic metastasis. Importantly, the levels of INHBA in CRC plasma were significantly increased compared with those in noncancer plasma. INHBA showed a higher level in dMMR/MSI CRC than in MSS CRC, indicating that INHBA is a useful biomarker.

**Conclusion:**

Our results showed that ONT-identified genes provide a pool to explore AS-associated markers for dMMR/MSI CRC. We demonstrated INHBA as a promising signature for clinical application in predicting tumor lymphatic metastasis and screening dMMR/MSI candidates.

## 1. Introduction

Colorectal cancer (CRC) is one of the most important malignant gastrointestinal tumors and has demonstrated high rates of mortality and morbidity [1]. CRC is classified as mismatch repair deficient (dMMR) or microsatellite instability (MSI) by the absence of at least one of four mismatch repair proteins (MLH1, MSH2, MSH6, or PMS2) [2]. The characteristics of MSI CRC include a high tumor mutational burden, generating hyperneoantigens that are responsible for favorable clinical responses to immune checkpoint blockade (ICB) [3]. However, approximately 10~15% of CRC cases display dMMR/MSI [4].

Currently, dMMR/MSI screening is helpful to select the most appropriate ICB for CRC and is also used as a biomarker to predict the therapeutic response to chemotherapy [5]. Patients with metastatic CRC (mCRC) that are high in dMMR/MSI are less responsive to conventional chemotherapy with or without drugs targeting VEGF or EGFR [6]. The KEYNOTE-177 study showed that pembrolizumab (anti-PD1) monotherapy provides a clinically meaningful improvement in progression-free survival (PFS) compared with chemotherapy for dMMR/MSI mCRC (median PFS: 16.5 months *vs.* 8.2 months) [7]. Additionally, mCRC patients with dMMR/MSI treated with first-line pembrolizumab showed better health-related quality of life than those treated with chemotherapy [2].

Cancer-associated mutations in genes encoding RNA alternative splicing (AS), such as MLH1, commonly occur in CRC [8]. AS is a rich source of tumor-specific neoantigen targets for ICB [9]. To some extent, MSI patients can benefit from ICB depending on large numbers of immunogenic neoantigens that can be recognized by immune cells [10]. Mutations in MMR proteins confer an increased lifetime risk of cancer development in affected individuals, while neoantigens result from the hypermutable nature of dMMR [11]. AS is partly responsible for transcript variation and proteome diversity [2]. MLH1 deficiency triggers chromosomal abnormalities and the release of nuclear DNA into the cytoplasm, resulting in the activation of the cyclic GMP-AMP synthase- (cGAS-) STING pathway, whose key molecules undergo AS [12]. cGAS acts as a specific pattern recognition receptor and is activated by type I interferons (IFNs), which mediate the Janus kinase- (JAK-) signal transducer and activator of transcription (STAT) pathway [13]. Through AS, STAT3 is translated into two distinct isoforms, STAT3*α* and STAT3*β* [14]. Therefore, identification dMMR-related AS genes would be helpful on either illustrating novel mechanism for ICB or screening biomarker for predicting therapeutic effect.

Comparing with microsatellite stability (MSS) CRCs, dMMR CRCs exhibited distinct immunological feature. Activation of cGAS-STING and type I IFN signaling promotes the overexpression of CCL5 and CXCL10 and recruitment CD8^+^ T cells into dMMR CRC TME [15]. CXCL10 is upregulated in homologous recombination-deficient tumors and binds to CXCR3 [16]. CXCR3 is primarily expressed on activated CD8^+^ T cells and is suppressed by transforming growth factor beta (TGF*β*) [17]. TGF*β* signaling is the major pathway that excludes immune cells from tumors. MSI CRC is unresponsive to TGF*β* because of TGFBR2 mutations [18]. Thus, different molecular patterns exist between MSI and MSS patients, but their identities remain elusive.

High-throughput sequencing technology has become increasingly available to identify differential mRNA profiles or genomic variations. Nanopore sequencing technology with highly accurate quantification of transcripts is a third-generation sequencing method recently developed to sequence full-length RNA transcripts as well as distinct AS transcripts [19]. In the present study, we performed transcriptome sequencing to survey the full-length CRC transcriptome landscape offered by Oxford Nanopore Technologies (ONT). We further compared the differential profiles between MSI and MSS tumors. Our data provide the first full-length MSI and MSS CRC transcriptome databases, allowing us to identify INHBA as biomarkers to discern among MSI CRC patients using blood plasma.

## 2. Materials and Methods

### 2.1. Patient Selection

For ONT RNA sequencing, 20 fresh samples were collected from 10 CRC patients from 2020 to 2021 at Beijing Chao-Yang Hospital. These CRC patients enrolled five dMMR/MSI patients. dMMR/MSI was defined when any of these MMR proteins were completely absent in the cancer tissue but presented in adjacent benign tissue. Microsatellite stability (MSS) patients were defined when all of MMR (MLH1, MSH2, MSH6, and PMS2) were positive expression in both tumor and adjacent benign tissue. Immunohistochemistry (IHC) was assayed to detect MLH1, MSH2, MSH6, and PMS2 separately. Patients who showed dMMR were defined as MSI. For validation, 23 paired tumor samples and tumor-adjacent normal fresh tissues were collected and stored at -80°C until to use. Forty-six normal intestinal tissues and 49 cancer samples were formalin-fixed and formalin-embedded and reassessed by two pathologists. Sixty-six peripheral plasma samples from CRC patients and 20 plasma samples from nontumor donors were separated and stored at -80°C until use. This study was approved by the Institutional Review Board at Beijing Chao-Yang Hospital. All the enrolled subjects signed written informed consent forms.

### 2.2. ONT Sequencing

One microgram of total RNA was prepared for cDNA libraries using a cDNA-PCR Sequencing Kit (SQK-PCS109; ONT). The products were then subjected to ONT adaptor ligation using T4 DNA ligase (NEB). The final cDNA libraries were added to FLO-MIN109 flow cells and run on the PromethION platform at Biomarker Technology Company (Beijing, China). Raw reads were first filtered (minimum average read quality score = 7 minimum read length = 500 bp). Ribosomal RNAs were discarded after mapping to the rRNA database. Clusters of full-length, nonchimeric (FLNC) transcripts and consensus isoforms were obtained after mapping to the reference genome (GRCh38_release95) with minimap2 (version 2.7-r654) in spliced alignment mode after polishing within each cluster using pinfish [20]. Mapped reads were further collapsed by the cDNA_Cup cake package with min‐coverage = 85%and min‐identity = 90%. The criteria for fusion candidates are that a single transcript must map to ≥2 loci (minimum coverage 5%, ≥1 bp; total coverage ≥ 95%, at least 10 kb). The transcripts were validated against known reference transcript annotations (hg38) with Gffcompare (version 0.1.26) [21]. Alternative splicing (AS) events, including IR (intro retention), ES (exon skipping), AA (Alternative acceptor), and MEE (mutually exclusive exon), were identified using the AStalavista tool (version 3.0) [22]. Simple Sequence Repeat (SSR) of the transcriptome were identified using MIcroSAtellite identification tool (version 2.1) [23]. Alternative polyadenylation (APA) analysis was conducted with TAPIS (Transcriptome Analysis Pipeline for Isoform Sequencing, version 1.2.1) [24]. Coding Sequence (CDS) were predicted by TransDecoder (version 5.5.0) [25]. The data were submitted to the NCBI SRA database and are accessible through the citation accession number PRJNA758110. For quantification, the expression levels were estimated by reads per gene/transcript per 10,000 reads mapped. Differential expression analysis was performed using the DESeq2 R package (version 1.6.3). These bioinformatic analyses were performed by Biomarker Technology Company. Genes with an adjusted *P* value < 0.01 and fold change (FC) ≤ −1.5 or ≥1.5 found by DESeq2 were assigned as differentially expressed. Gene Ontology (GO) enrichment analysis of the differentially expressed genes (DEGs) was implemented using the DAVID Bioinformatics Resources (version 6.8) tool [26] and plotted using GOplot R packages (version 1.0.2) [27]. GO terms included biological process (BP), cellular component (CC), and molecular function (MF). The top eight terms of biological process (BP) were statistically analyzed according to *P* value (<0.05) and FC ≤ −1.5 or ≥1.5.

### 2.3. TCGA Dataset Validation

COAD (enrolled 41 normal tissue samples and 457 cancer tissue samples) and READ (enrolled 10 normal tissue samples and 166 cancer tissue samples) datasets were downloaded from The Cancer Genome Atlas (TCGA) data portal (http://www.cbioportal.org/). The R package “limma” [28] was used to screen for the differentially expressed genes between cancer and normal samples according to the criteria of an adjusted *P* value < 0.01 and fold change (FC) ≥ ≤−1.5 or ≥1.5.

### 2.4. Estimating the Proportion of Immune and Cancer (EPIC) and CIBERSORT Analysis

EPIC and CIBERSORT were used to estimate the fraction of immune cells of sequenced samples. EPIC was performed using an online tool (http://epic.gfellerlab.org/). The R package “CIBERSORT” (R script version 1.03) was applied to assess the fractions of the 22 types of tumor-infiltrating immune cells [29]. Outputs were considered to be accurate after screening to meet *P* value < 0.05.

### 2.5. Reverse Transcription Quantitative Real-Time PCR (RT-qPCR)

Total RNA was purified from 46 samples using TRIzol Reagent (Invitrogen, CA, USA). A QuantiTect Reverse Transcription kit (Qiagen, Germany) was used for reverse transcription. An ABI7500 Real-Time PCR System (Applied Biosystems, USA) was used to detect the expression of candidate genes. The primers verified in this study are shown as follows:

1. INHBA: forward—5′-CAGTGCCAATACCATGAAGAGGA-3′; reverse—5′-ATGCAAAACTAGGGAAGAGAACCC-3′
2. RPL22L1: forward—5′-AAAGACAGGAAGCCCAAGAGG-3′; reverse—5′-AGGGCATCCTGGCAGCAAAA-3′
3. CAPZA1: forward—5′-CGAATGAAGCCCAAACTGCC-3′; reverse—5′-TTTGGTGCGGGTAACTGGAA-3′
4. HMGCS2: forward—5′-AGTGGTAATGCTCGTCCCAC-3′; reverse—5′-ATATGGGTTCCCCTCAGCCC-3′
5. 18S (reference gene): forward—5′-AAACGGCTACCACATCCA-3′; reverse—5′-CACCAGACTTGCCCTCCA-3′

### 2.6. IHC Assays

The primary antibodies used in IHC for tissue slides were as follows: anti-INHBA (Proteintech; #10651-1-AP; 1 : 50 dilution), anti-RPL22L1 (Cusabio; #CSB-PA740724A01HU; 1 : 50 dilution), and anti-CAPZA1 (CSB-PA004510LA01HU; 1 : 50 dilution). The slice was deparaffinized and rehydrated, pretreated with citric acid antigen retrieval solution (pH = 6.8), and rinsed in PBS. The sections were then blocked in 2% goat serum and incubated with the primary antibody overnight at 4°C. The streptavidin-peroxidase method (ZSGG-BIO, China; #PV9001) was used to show the levels of stained proteins. The percentage of positive cells was scored as follows: 1 (0–25%), 2 (26–50%), 3 (51–75%), and 4 (>75%). The intensity of positive staining was classified into 4 scales as follows: 0 (negative), 1 (weak), 2 (moderate), and 3 (strong). The levels were semiquantitatively determined as percentages multiplied by intensity.

### 2.7. Enzyme-Linked Immunosorbent Assay (ELISA)

The amount of INHBA in blood plasma was quantified using the Human INHBA ELISA Kit (#ml022686; mlbio, China) according to the manufacturer's instructions. Briefly, 50 *μ*l of plasma was added to 96-well anti-INHBA-coated plates and incubated at 4°C overnight. After that, the samples were removed and washed with washing buffer, and then horseradish peroxidase-labeled antibodies were added and incubated at 37°C for 1 hour. Finally, substrate solution was added and terminated by adding stop solution. Each well was measured at an Optical Density (O.D.) of 450 nm using a microtiter plate reader within 15 minutes. The INHBA concentrations were calculated using a standard curve.

### 2.8. Statistical Analysis

We used unpaired Student's *t*-test and the Mann–Whitney test as indicated, and binary outcomes were compared. A *P* value less than 0.05 was considered statistically significant. Analysis was performed using GraphPad Prism software (version 8.0.1).

## 3. Results

### 3.1. Long Read RNA Sequencing Establishes CRC-Related Differential Profiles

**Figure 1:**
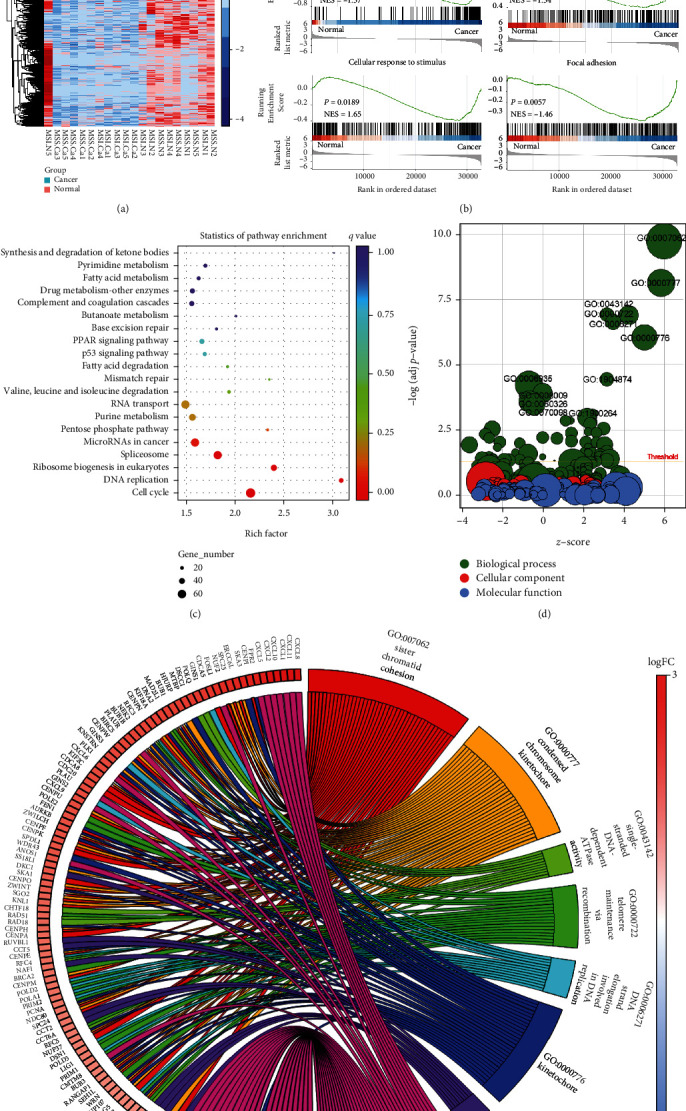
Differential mRNA profiles of CRC ONT sequencing reveal the most dysregulated immune-related pathways and genes. (a) Heatmap clusters of the mRNA levels of 4,179 genes in 20 samples. (b) GSEA enriches the pathways for differentially expressed genes between cancer and normal tissues. (c) KEGG analysis investigates the pathway for these differential genes involved. (d). Gene Ontology (GO) identifies the biological process (BP), cellular component (CC), and molecular function (MF) for differentially expressed genes. (e) The top eight BP terms are plotted, and the genes enriched in these terms are shown with fold changes.

To investigate the differential mRNA profiles of CRC, we used full-length RNA sequencing by ONT, which is a third-generation RNA-seq technique, and constructed transcriptome landscapes in 20 CRC samples, which were paired normal and cancer tissues (Figure S1, Figure S2, Supplementary Table S1). ONT sequencing identified 8,566 transcripts with fold changes ≤ −1.5 or ≥1.5 and a *P* value < 0.01, encoding 4,179 genes (Figure 1(a)). The downregulated genes numbered 2,632, and the upregulated genes numbered 1,547 in cancer tissues. Gene set enrichment analysis (GSEA) indicated that these genes are involved in the primary immunodeficiency, regulation of actin cytoskeleton, cellular response to stimulus, and focal adhesion (Figure 1(b)). Additionally, pathway enrichment analysis demonstrated that these genes were significantly enriched in DNA replication and cell cycle pathways (Figure 1(c)). To explore the biological function, Gene Ontology (GO) enrichment analysis was performed, and the eight top GO terms associated with immune response and DNA replication were enriched (Figures 1(d) and 1(e)). Several chemokines enriched in chemotaxis terms, including CXCL8/11/1/10/5, were significantly increased in cancer tissues (Figure 1(e)). Our GO analyses enriched many genes associated with sister chromatid cohesion and condensed chromosome kinetochores, which are involved in DNA replication, one of the most important preconditions for the cell cycle (Figure 1(e)).

To validate our findings, we identified differential mRNAs in TCGA colorectal cancer datasets (COAD and READ) (Figure S3A). In total, 5,121 and 4,924 differentially expressed genes were identified in both datasets. Additionally, 1,417 overlapping genes showed differential expression by either ONT sequencing or TCGA analysis (Figure S3B). GO analysis was also used to enrich the biological processes for COAD differential genes, indicating that these genes were involved in GO terms concerning the chemokine-mediated signaling pathway, cellular response to tumor necrosis factor, and cellular response to interleukin-1 (Figure S3C). For READ differential genes, GO terms concerning the chemokine-mediated signaling pathway, cell chemotaxis, and chemotaxis were enriched as the most important biological processes for these genes involved (Figure S3C). The above results showed that chemokines play a key role in regulating CRC progression.

### 3.2. dMMR/MSI Related Genes

**Figure 2:**
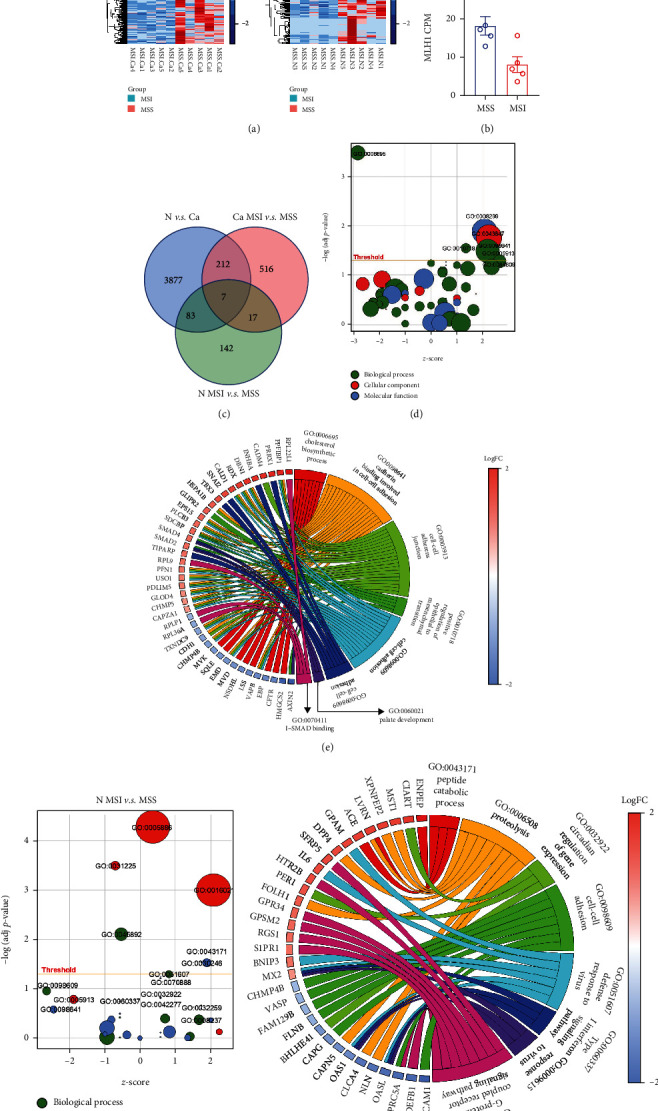
Identification of dMMR/MSI-related genes between cancer and normal CRC tissues. (a) Heatmap illustrating the differentially expressed genes in dMMR/MSI and MSS CRC. The dysregulated genes are clustered in cancer (left) and normal (right) tissues. (b) MLH1 is the marker for dMMR, and its levels were compared between MSS (*n* = 5) and MSI (*n* = 5) tissues using ONT sequencing data. (c) Venn diagram analysis of the significant overlap of upregulated or downregulated genes between dMMR/MSI and MSS and comparing these genes with those showing differential levels in cancer (Ca) or normal (N) CRC tissues. (d) GO analysis enriches dMMR/MSI-related genes in cancer tissues. (e) Circos plot displaying the GO BP terms and dMMR/MSS-related genes. (f) GO enrichment shows the genes identified in dMRR/MSI normal tissues. (g) Circos plot delineates the GO BP terms and their enriched genes.

We further addressed the differentially expressed genes between MSI and MSS to screen the biomarkers associated with dMMR. ONT sequencing analyzed five MSIs and five MSSs. In cancer tissues, 752 differentially expressed genes were identified; 404 genes were downregulated, and 348 genes were upregulated in MSI cancer tissues compared with MSS cancer tissues (Figure 2(a)). In normal tissues, 249 differential genes were identified in MSI, comprising 126 upregulated genes and 123 downregulated genes (Figure 2(a)). The MLH1 gene is a hallmark of dMMR and was significantly decreased in MSI cancer tissues in our ONT analysis (Figure 2(b)). Two hundred thirty-six overlapping genes with differential expression were identified when comparing cancer-MSI versus (*vs.*) cancer-MSS or cancer *vs.* cancer tissue (Figure 2(c)). Additionally, 107 overlapping genes were detected when comparing normal-MSI *vs.* normal-MSS or cancer *vs.* normal tissues (Figure 2(c)).

To assess the biological features of these differentially expressed genes, we employed GO analysis to feature the terms for MSI-related genes. In cancer tissues, the most significant GO terms for MSI-specific genes included GO:0006695 (cholesterol biosynthetic), GO:0008299 (isoprenoid biosynthetic), and GO:0098609 (cell-cell adhesion), which referred to biological process, molecular functions, and cellular component, respectively (Figure 2(d)). In normal tissues, the most significant GO terms were GO:0005886 (plasma membrane) and GO:0045892 (negative regulation of transcription, DNA–templated), which were classified as cellular component and biological process, respectively (Figure 2(f)). Additionally, both cancer and normal tissues MSI-related differential genes showed similar GO terms, and most of these genes were involved in metabolic-related processes, such as cholesterol biosynthetic and peptide catabolic process (Figures 2(e) and 2(g)).

### 3.3. Exploration of MSI-Related Alternative Splice (AS) Events

**Figure 3:**
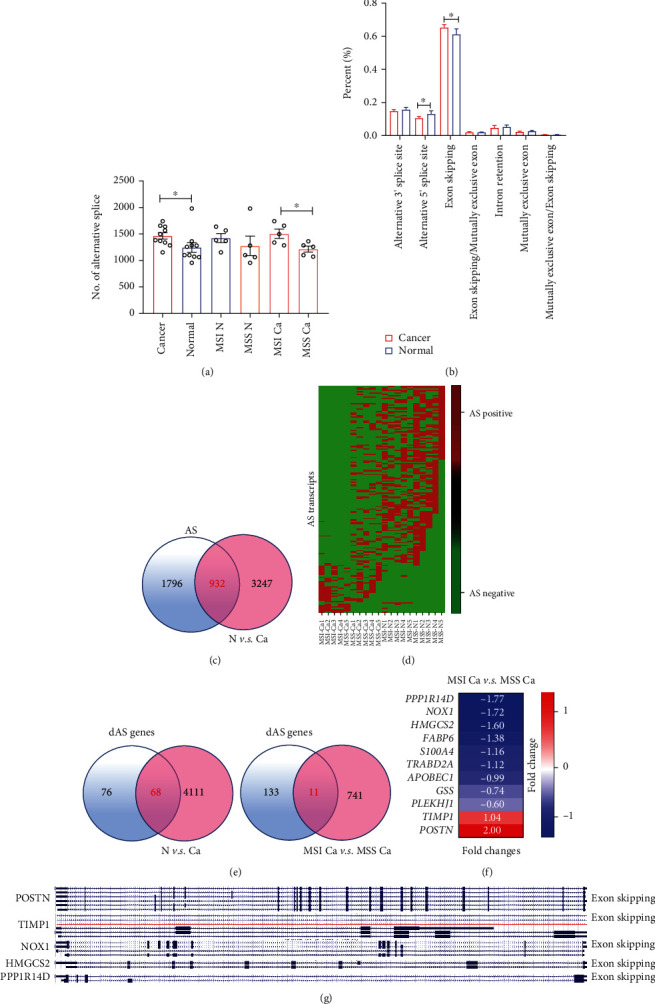
Alternative splicing (AS) is significantly associated with dMMR CRC. (a) The number (no.) of AS-related events was compared in cancer *vs.* normal controls. Normal tissue, MSI N *vs.* MSS N, and MSI Ca. *vs.* MSS Ca. (b) The percentages of seven AS type forms were compared between cancer and normal tissues. (c) Venn diagrams representing the overlapping genes that were differentially expressed and edited by AS. (d) Screening of AS-edited genes specifically associated with dMMR/MSI. Rows represent alternative splicing transcripts. E. Venn diagrams of dMMR/MSI-specifically related AS- (dAS-) edited genes as well as differentially expressed genes between cancer and normal tissues (left) and genes differentially upregulated or downregulated in MSI (right). (f) Eleven AS edited genes are listed with fold changes. (g) Graphical depiction of ENSEMBL transcripts from POSTN, TIMP1, NOX1, HMGCS2, and PPP1R14D. *P* values were obtained by nonparametric Mann–Whitney test (a, b) data are depicted by bar chart with error ± standard deviation of mean (SEM). ^∗^*P* < 0.05.

To identify AS landscape, these transcripts determined using ONT sequencing were aligned to against known reference transcript annotations (hg38, UCSC) [30]. This analysis allows identification of the following 7 types of alternative splice events: (1) alternative 3′ splice site, (2) alternative 5′ splice site, (3) exon skipping, (4) exon skipping/mutually exclusive exon, (5) intron retention, (6) mutually exclusive exon, and (7) mutually exclusive exon/exon skipping. In total, ONT sequencing detected 6,200 AS events in 20 samples, which were found in 2,728 genes. The frequencies of AS were significantly higher in cancer tissues than in normal tissues, indicating that AS-related variations play critical roles in regulating tumor progression (Figure 3(a)). Additionally, AS frequencies were significantly increased in MSI compared with MSS and revealed that AS might be the signature for dMMR (Figure 3(a)). Exon skipping was the most important AS event, accounting for nearly 60% of the total AS, and was significantly increased in cancer tissues (Figure 3(b)). However, alternative 5′ splice sites were significantly decreased in cancer tissues (Figure 3(b)). In total, 932 genes with AS events were significantly differentially expressed in cancer tissues (Figure 3(c)). To screen AS events specifically expressed in MSI, we selected AS events that occurred in three or more of these 20 samples, as a result, 170 MSI-related AS transcripts were produced which were transcribed by 144 genes (Figure 3(d)). Among 144 genes, 68 genes were differentially expressed when comparing their levels between cancer and normal tissues (Figure 3(e)). Eleven AS-edited genes had differential levels when compared their expression in MSI with their levels in MSS (Figures 3(e) and 3(f)). PPP1R14D, NOX1, and HMGCS2 were downregulated, while POSTN and TIMP1 were upregulated in MSI tissues (Figure 3(f)). According to UCSC analysis [31], both POSTN and TIMP1 had six transcripts and were transcribed through exon skipping, and NOX1 was encoded by four transcripts while transcribed by exon skipping. HMGCS2 and PPP1R14D were edited by exon skipping to transcribe two types of transcripts (Figure 3(g)).

### 3.4. Infiltrated Immune Cells in Tumor Microenvironment Were Identified by EPIC and CIBERSORT Analysis

Estimating the proportion of immune and cancer (EPIC) and CIBERSORT are designed to identify infiltrated immune cells and cancer cells from bulk RNA sequencing. Both tools are used to assess the proportion of immune cells and cancer cells in ONT sequencing data. EPIC identified 8 types of cells in 20 samples (Figure S4A), and immune cells, including CD8 T cells, NK cells, and B cells, were higher in normal tissues than in cancer tissues (Figure S4B). By contrast, endothelial cells and macrophages were decreased in cancer tissues (Figure S4B). Cancer-associated fibroblasts (CAFs) and NK cells were significantly increased in MSI (Figure S4C). CIBERSORT recognized 22 cell types, which included several subtypes of immune cells. Plasma cells accounted for the highest proportion in our ONT data (Figure S4D), and these cells were decreased in cancer tissues (Figure S4E). Additionally, activated mast cells, activated/resting dendritic cells, activated NK cells, and naïve B cells were increased, while M2 macrophages and resting CD4 memory cells were decreased in cancer tissues (Figure S4E). In CIBERSORT analysis, activated mast cells were abundant in MSI cancer (Figure S4F). EPIC and CIBERSORT analysis indicated significantly differential immune cell patterns between cancer and normal tissues.

### 3.5. WGCNA Identifies MSI-Specific Clusters

**Figure 4:**
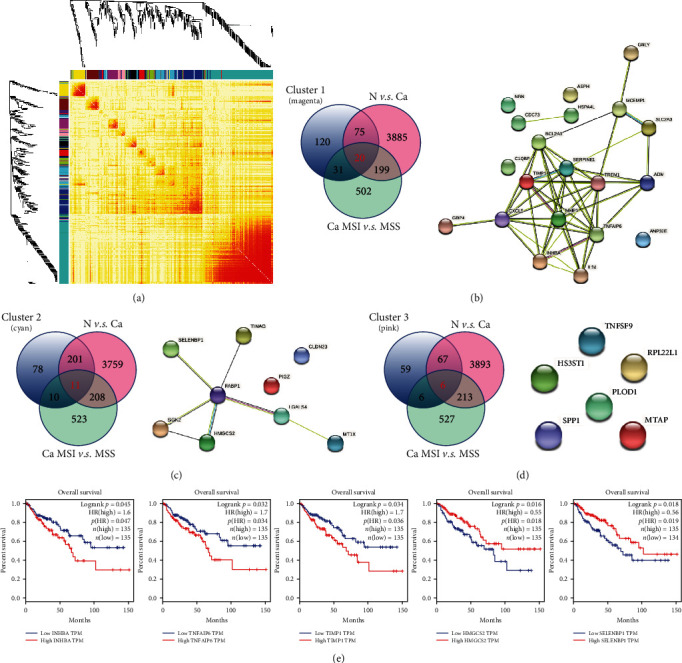
dMMR/MSS-related gene modules mined through weighted gene coexpression network analysis (WGCNA). (a) WGCNA analyzes ONT sequencing data. Gene clustering tree (dendrogram), module colors (main modules and submodules), and labels. (b) WGCNA identifies cluster 1, which contained 20 genes with differential levels either comparing cancer and normal samples or comparing dMMR/MSS and MSS samples (left). The protein-protein networks (PPIs) were investigated using the STRING tool (https://www.string-db.org/) (right). (c) Cluster 2 contained 11 genes (left) used to construct the PPI (right). (d) Cluster 3 included 6 genes (left) that showed no interactive relationship (right). (e). The clustered genes were associated with overall survival (OS) rates using TCGA COAD datasets according to GEPIA (http://gepia.cancer-pku.cn/).

Weighted gene coexpression network analysis (WGCNA) was performed across all the sequencing samples to address the specific clusters associated with MSI. In total, these genes can be divided into 17 clusters, which are indicated with different colors in Figure 4(a). Three clusters showed differential expression in MSI that might represent signatures. Cluster 1 included 246 genes; among them, 20 overlapped genes were simultaneously shown to be differentially expressed in MSI and cancer *vs.* normal tissues (Figure 4(b)). INHBA, MMP1, and SERPINE1 comprised a protein-protein interacting (PPI) network according to STRING network analysis, and these genes were significantly increased in cancer tissues or MSI cancer tissues (Figure 4(b)). In cluster 2, HMGCS2, FABP1, and SELENBP1 were significantly decreased in MSI according ONT sequencing result, consistent with an interacting network (Figure 4(c)). Cluster 3 included several upregulated genes in MSI and failed to enrich any PPI network (Figure 4(d)). We further addressed the overall survival (OS) relevant to the TCGA dataset using the GEPIA tool. The results demonstrated that the overexpression of INHBA, TNFAIP6, and TIMP1 was associated with a lower OS rate (Figure 4(e)). By contrast, increases in HMGCS2 and SELENBP1 were associated with a higher OS rate (Figure 4(e)).

### 3.6. Validation of MSI-Associated Genes

**Figure 5:**
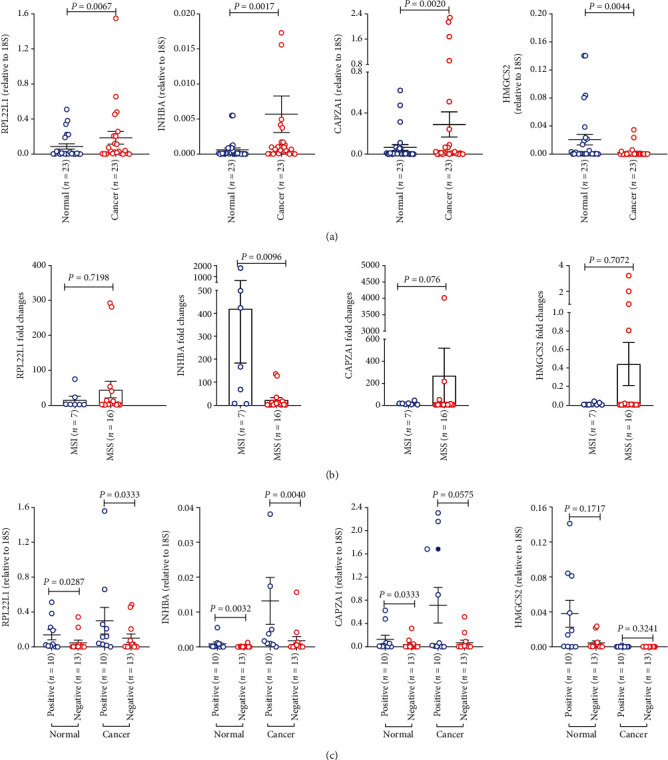
RT-qPCR assays validate the differential expression of selected genes. (a) RPL22L1, INHBA, and CAPZA1 are increased in cancer tissues; conversely, HMGCS2 is decreased. (b) The fold changes of INHBA are significantly upregulated in MSI compared with those in MSS. (c) RPL22L1 and INHBA expression are associated with tumor lymphatic metastasis. *P* values were obtained by paired Student's *t*-test (a) and nonparametric Mann–Whitney test (b, c); data are depicted by scatter plots (a, c) with mean ± SEM or bar chart and scatter plots (b) with error ± SEM.

Our ONT sequencing combined tremendous information in CRC, particularly for MSI, allows identification of biomarkers to screen CRC or MSI candidates. As mentioned above, the third-generation sequencing technique allowed us to perform integrative analysis of AS and gene levels at the RNA level. Our GO enrichment revealed that RPL22L1, INHBA, and CAPZA1 had a significantly higher expression levels in MSI than in MSS, and these genes were also upregulated in CRC cancer tissues compared with their levels in matched normal tissues. RPL22L1 transcripts were edited by alternative 3′ splicing and were identified in all 20 samples. HMGCS2 was downregulated in CRC cancer tissue and showed lower levels in MSI than in MSS. Additionally, HMGCS2 is an AS transcript that is not expressed in MSI and serves as a signature for MSI. Importantly, RPL22L1, INHBA, and HMGCS2 were involved in the I-SMAD binding biological process (Figure 2(e)). Therefore, we selected these four genes to further explore their differential expression using RT-qPCR in 23 paired cancer and normal tissues. As expected, the mRNA levels of RPL22L1, INHBA, and CAPZA1 were significantly higher in cancer than in normal tissues; conversely, HMGCS2 was significantly downregulated in cancer (Figure 5(a)). Next, we questioned whether these genes could be signatures for MSI. We compared their fold changes between MSI and MSS, and INHBA showed significantly increased levels in MSI (Figure 5(b)). In cancer tissues, RPL22L1 and INHBA were significantly upregulated in CRC with positive lymph nodes. In normal tissues, RPL22L1, INHBA, and CAPZA1 were found to be significantly increased in CRC patients for whom experienced tumor lymphatic metastasis (Figure 5(c)).

**Figure 6:**
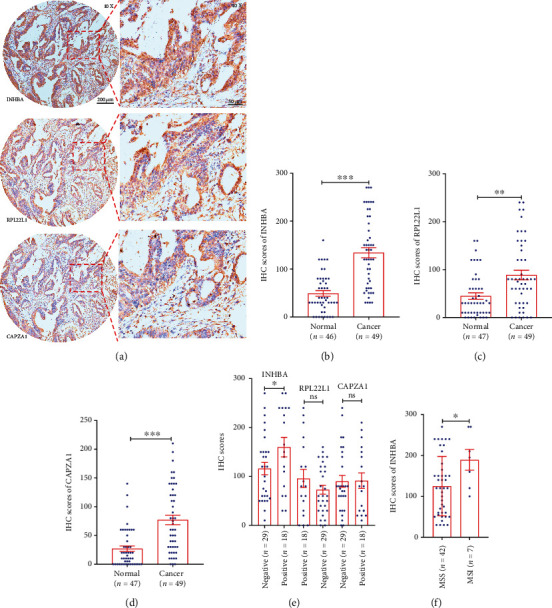
Immunohistochemistry (IHC) assays determine the overexpression of screened genes. (a) Representative IHC pictures show the expression of INHBA, RPL22L1, and CAPZA1 in tissue splices obtained from the same sample. (b) Comparison of the levels of INHBA validates its overexpression in cancer tissues. (c) RPL22L1 levels were compared in cancer and normal tissues. (d) CAPZA1 shows higher levels in cancer than in normal tissues. (e) The levels of INHBA, RPL22L1, and CAPZA1 were determined in lymph node-positive metastatic tumors and lymph node-negative metastatic tumors (two patients missing information). (f) INHBA expression was significantly increased in dMMR/MSI cancer. *P* values were obtained by nonparametric Mann–Whitney test (b–f), data are depicted by bar chart and scatter plots (b–f) with error ± SEM. ^∗^*P* < 0.05,  ^∗∗^*P* < 0.01, and^∗∗∗^*P* < 0.001; ns: not significant.

Next, we applied IHC to determine the protein levels of INHBA, RPL22L1, and CAPZA1. Forty-nine cancer tissues and 47 normal tissues were enrolled for IHC analysis. Their levels were scored using semiquantitative analysis, demonstrating that these three genes were significantly overexpressed in cancer tissues (Figures 6(a)–6(d)). IHC assays were consistent with our RT-qPCR analyses. We also addressed clinicopathological relevance and found that INHBA was significantly increased in patients diagnosed with lymph node metastasis tumors (Figure 6(e)). INHBA shown higher levels in male CRC patients than their levels in female (Table S2). Other clinicopathological factors included tumor size, differentiation, TNM stage, and vascular and nerve invasion; INHBA levels did not shown statistic differences (Tables S2). We inferred based on both mRNA and protein that INHBA expression can be used as a biomarker to predict tumor lymphatic metastasis. Seven patients who were enrolled in the IHC analysis were diagnosed with MSI. We compared the expression of INHBA in these patients and found significantly higher levels in patients with MSI than in MSS patients (Figure 6(f)).

### 3.7. INHBA Is a Signature for MSI Diagnosis in Blood Plasma

**Figure 7:**
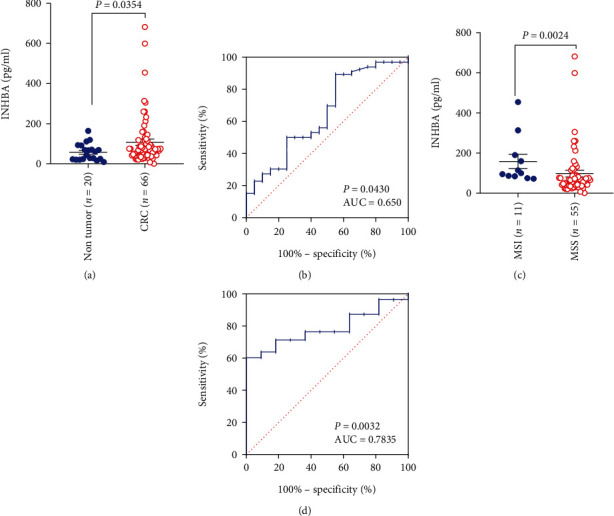
The detection of INHBA in blood plasma identifies a biomarker for CRC. (a) In plasma, the levels of INHBA were significantly increased in patients with CRC. (b) Receiver operating characteristic (ROC) curve analysis of the diagnostic efficacy. (c) INHBA levels in dMMR/MSI patients were higher than those in MSS patients. (d) INHBA is a biomarker for diagnosing dMMR/MSI candidates. *P* values were obtained by nonparametric Mann–Whitney test (a, c); ROC curves were plotted using GraphPad Prism software to calculate *P* and area under the curve (AUC) (b, d). Data are depicted by scatter plots (a, c) with mean value ± SEM.

Because INHBA is a protein secreted into the extracellular space by tumor cells, we applied ELISA to determine its plasma levels. Our analysis enrolled 20 plasma samples from patients diagnosed with nontumor benign disease and 66 plasma samples from CRC patients. As expected, the CRC plasma levels of INHBA were significantly increased compared with those of samples from nontumor benign disease patients (Figure 7(a)). Receiver operating characteristic (ROC) curve analysis was performed to examine the diagnostic efficiency when INHBA was recognized as a biomarker. Area under the curve (AUC) analysis indicated that secreted INHBA was a promising biomarker that could be used in clinical practice (AUC = 0.650; *P* = 0.0430) (Figure 7(b)). We further examined whether secreted INHBA served as a marker to discern dMMR/MSI CRCs. As expected, the levels of INHBA in dMMR/MSI patients were significantly higher than those in patients with MSS (Figure 7(c)). ROC curve analysis proved that INHBA could diagnose dMMR/MSI CRCs (AUC = 0.7835; *P* = 0.0032) (Figure 7(d)).

## 4. Discussion

In the present study, we constructed CRC mRNA profiles using ONT long read sequencing, which has a unique advantage in identifying the AS of mRNA transcripts. dMMR/MSI tumors exhibit a high frequency of AS, which is associated with a large burden of destabilizing mutations [32]. Utilizing integrative bioinformatic analysis, we demonstrated that immune chemotactic genes are the most differentially expressed in cancer tissues and mediate the recruitment of immune cells [33]. Both B and T cells are the major class of immune cells in the adaptive immune system and have multiple functions in facilitating the coevolution of cancer and its microenvironment [34]. Our ONT data consistently indicated that these cells are decreased in cancer tissues. Innate immune cells, including NK cells, macrophages, and mast cells, were dysregulated and showed differential levels in MSI-related CRC. These findings are associated with the characteristics of innate cells, which infiltrate the microenvironment and display tumor phagocytosis or promote tumor growth [35].

Despite extensive investigation of genomic mutations and many genetic drivers, few studies have focused on AS in dMMR/MSI [36]. Here, despite using a small cohort, our ONT sequencing determined thousands of differentially expressed genes and AS events, which showed a higher efficacy than next-generation sequencing. Tumor mutational burdens predict the response efficiency to ICB treatment in dMMR/MSI tumors [37]. In our study, AS of mRNA transcripts was significantly correlated with dMMR/MSI and we screened several potential biomarkers to validate in CRC tissues or plasma. INHBA, RPL22L1, CAPZA1, and HMGCS2 showed significant differential levels in dMMR/MSI cancer tissues and were finally selected for further detection. RPL22L1, a ribosomal protein, is overexpressed in ovarian cancer and promotes cancer cell metastasis via the epithelial-to-mesenchymal transition [38]. Consistently, we validated its overexpression in CRC and found that it was associated with dMMR/MSI or tumor lymphatic metastasis. CAPZA1 regulates actin polymerization, which controls cell motility by binding to the barbed ends of actin filaments [39]. CAPZA1 is upregulated in hepatocellular carcinoma [40] and gastric cancer [39]. HMGCS2 is downregulated in CRC, serving as a critical downstream target of SLC38A4 to regulate tumor cell metabolism [41].

INHBA is a susceptibility gene for pancreatic cancer [42] that is involved in the TGF-*β* signaling pathway and was edited by AS in our ONT analysis. INHBA is associated with the CRC OS rate and plays a role in promoting tumor cell proliferation and migration [43]. We demonstrated that INHBA is significantly increased in CRC cancer cells and, more importantly, is a potential biomarker for discerning dMMR/MSI candidates.

The most important concept conveyed in this study is a comprehensive investigation of mRNA profiles using third-generation sequencing, which allows us to explore AS events related to CRC dMMR/MSI for the first time. These newly constructed ONT data identified multiple genes that could be applied as markers associated with CRC progression. We surveyed their potential clinical applications in small sample sizes, such as INHBA, a promising dMMR/MSI biomarker. Two major limitations of our study are its retrospective nature and single-center design. Further study is required to address the clinical relevance of INHBA or other identified genes as markers for MSI-H/dMMR solid tumors in a larger cohort.

## Data Availability

The data were submitted to the NCBI SRA database and are accessible through the citation accession number PRJNA758110.

## Supplementary Materials

Supplementary Materials Figure S1: a schematic diagram illustrates how samples were selected 1 and ONT sequencing carried out. Figure S2: hematoxylin-eosin staining shows normal tissue, MSS cancer tissue, and MSI cancer tissue. Figure S3: TCGA COAD and READ analysis validate the identified differential genes using ONT sequencing. A. Volcano plots showing the upregulated or downregulated genes in the COAD (left) and READ (right) datasets. B. Venn diagram determines the overlapping dysregulated genes using ONT sequencing detection as well as validation in COAD and READ. C. GO enrichment enriched BP terms for COAD (left) and READ (right). Figure S4: cell types in the tumor microenvironment (TME) are analyzed using EPIC and CIBERSORT tools. A. EPIC analysis shows the cell fractions. B. Comparison of the distributions per cell type in cancer and normal tissues was performed by EPIC. C. dMMR/MSI tumor has higher CAF and NK cells. D. Cell compositions in the TME were calculated using the CIBERSORT method. E. Cell compositions analyzed by CIBERSORT were compared between cancer and normal TMEs. F. The compositions of activated mast cells are increased in MSI. *P* values were obtained by nonparametric Mann–Whitney test (B, C, E); data are depicted by box and scatter plots (B, C, E) with whiskers (Min–Max). ^∗^*P* < 0.05,  ^∗∗^*P* < 0.01, and^∗∗∗^*P* < 0.001; ns: not significant.
