# Supplementary material for: Long-Read Nanopore Sequencing Identifies Mismatch Repair-Deficient Related Genes with Alternative Splicing in Colorectal Cancer
Source: Dis Markers. 2022 Jul 21;2022:4433270. doi: 10.1155/2022/4433270 (PMC9334049; doi:10.1155/2022/4433270)
Supplement: Supplementary Materials — Figure S1: a schematic diagram illustrates how samples were selected 1 and ONT sequencing carried out. Figure S2: hematoxylin-eosin staining shows normal tissue, MSS cancer tissue, and MSI cancer tissue. Figure S3: TCGA COAD and READ analysis validate the identified differential genes using ONT sequencing. A. Volcano plots showing the upregulated or downregulated genes in the COAD (left) and READ (right) datasets. B. Venn diagram determines the overlapping dysregulated genes using ONT sequencing detection as well as validation in COAD and READ. C. GO enrichment enriched BP terms for COAD (left) and READ (right). Figure S4: cell types in the tumor microenvironment (TME) are analyzed using EPIC and CIBERSORT tools. A. EPIC analysis shows the cell fractions. B. Comparison of the distributions per cell type in cancer and normal tissues was performed by EPIC. C. dMMR/MSI tumor has higher CAF and NK cells. D. Cell compositions in the TME were calculated using the CIBERSORT method. E. Cell compositions analyzed by CIBERSORT were compared between cancer and normal TMEs. F. The compositions of activated mast cells are increased in MSI. P values were obtained by nonparametric Mann–Whitney test (B, C, E); data are depicted by box and scatter plots (B, C, E) with whiskers (Min–Max). ∗P < 0.05, ∗∗P < 0.01, and∗∗∗P < 0.001; ns: not significant. [file 4433270.f1.pdf]

Figure S1 A schematic diagram illustrates how samples were selected and ONT sequencing carried out.

Figure S2 Hematoxylin-Eosin staining shows normal tissue, MSS cancer tissue, and MSI cancer tissue.

Figure S3 TCGA COAD and READ analysis validate the identified differential genes using ONT sequencing. A. Volcano plots showing the upregulated or downregulated genes in the COAD (left) and READ (right) datasets. B. Venn diagram determines the overlapping dysregulated genes using ONT sequencing detection as well as validation in COAD and READ. C. GO enrichment enriched BP terms for COAD (left) and READ (right).

Figure S4 Cell types in the tumor microenvironment (TME) are analyzed using EPIC and CIBERSORT tools. A. EPIC analysis shows the cell fractions. B. Comparison of the distributions per cell type in cancer and normal tissues was performed by EPIC. C. dMMR/MSI tumor has higher CAF and NK cells. D. Cell compositions in the TME were calculated using the CIBERSORT method. E. Cell compositions analyzed by CIBERSORT were compared between cancer and normal TMEs. F. The compositions of activated mast cells are increased in MSI.

P values were obtained by nonparametric Mann-Whitney test (B, C, E), data are depicted by box and scatter plots (B, C, E) with whiskers (Min to Max.). *$P<0.05$, **$P<0.01$, ***$P<0.001$; ns, not significant.

Figure S1

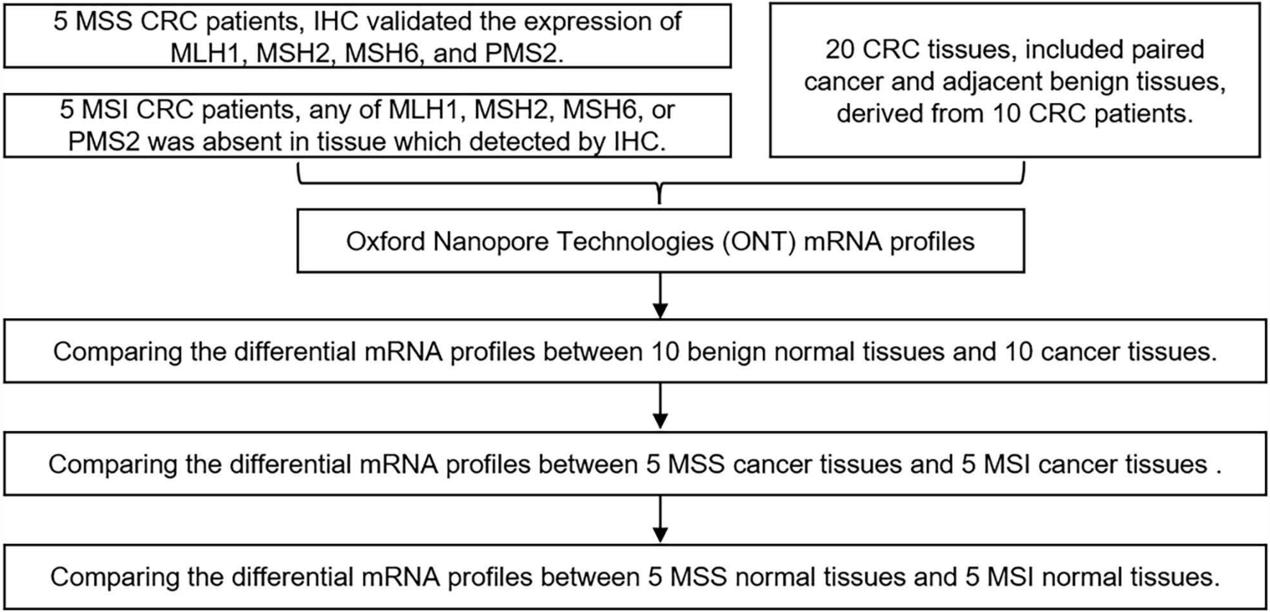
5 MSS CRC patients, IHC validated the expression of MLH1, MSH2, MSH6, and PMS2.
5 MSI CRC patients, any of MLH1, MSH2, MSH6, or PMS2 was absent in tissue which detected by IHC.
20 CRC tissues, included paired cancer and adjacent benign tissues, derived from 10 CRC patients.
Oxford Nanopore Technologies (ONT) mRNA profiles
Comparing the differential mRNA profiles between 10 benign normal tissues and 10 cancer tissues.
Comparing the differential mRNA profiles between 5 MSS cancer tissues and 5 MSI cancer tissues .
Comparing the differential mRNA profiles between 5 MSS normal tissues and 5 MSI normal tissues.

Figure S2

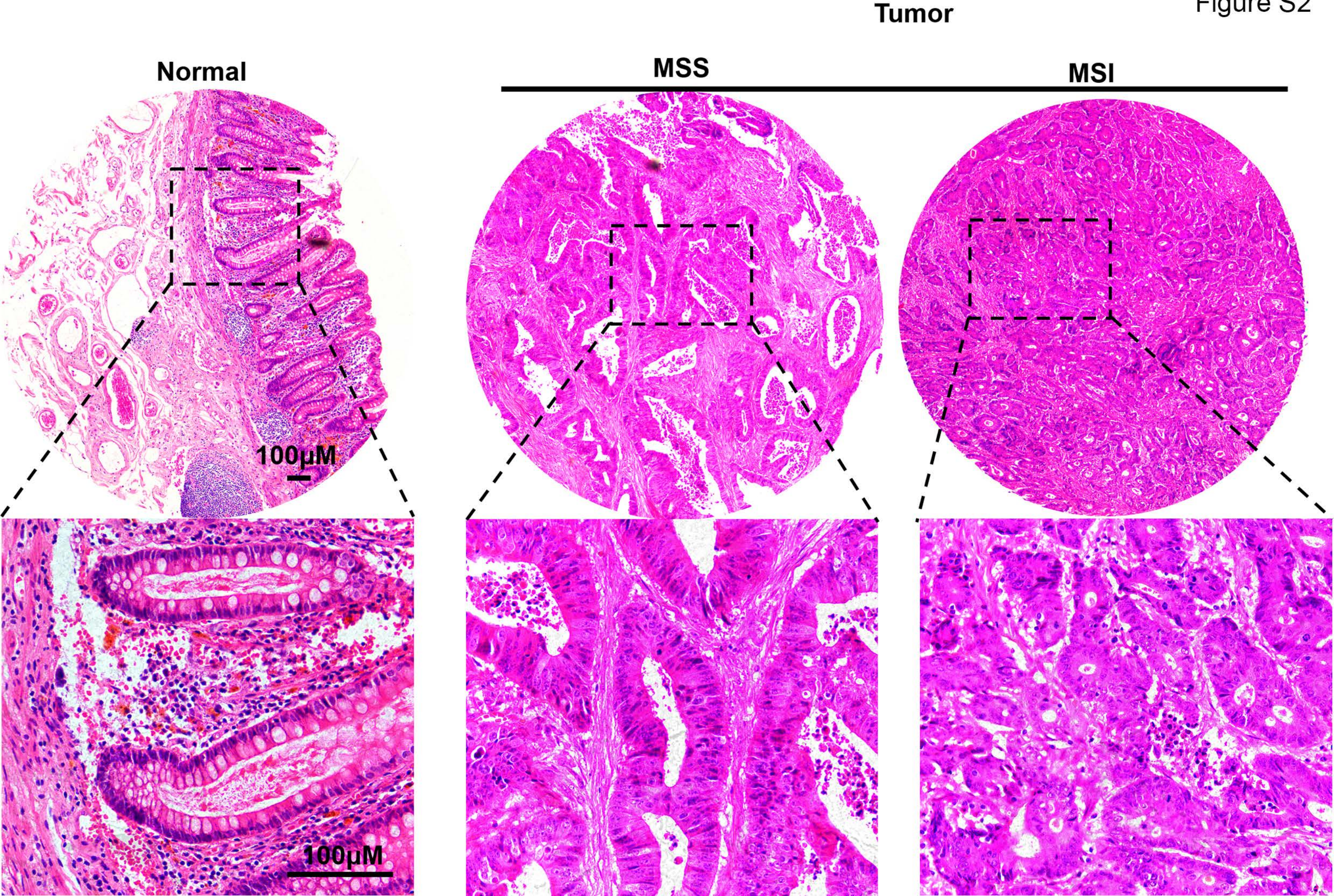

Figure S3

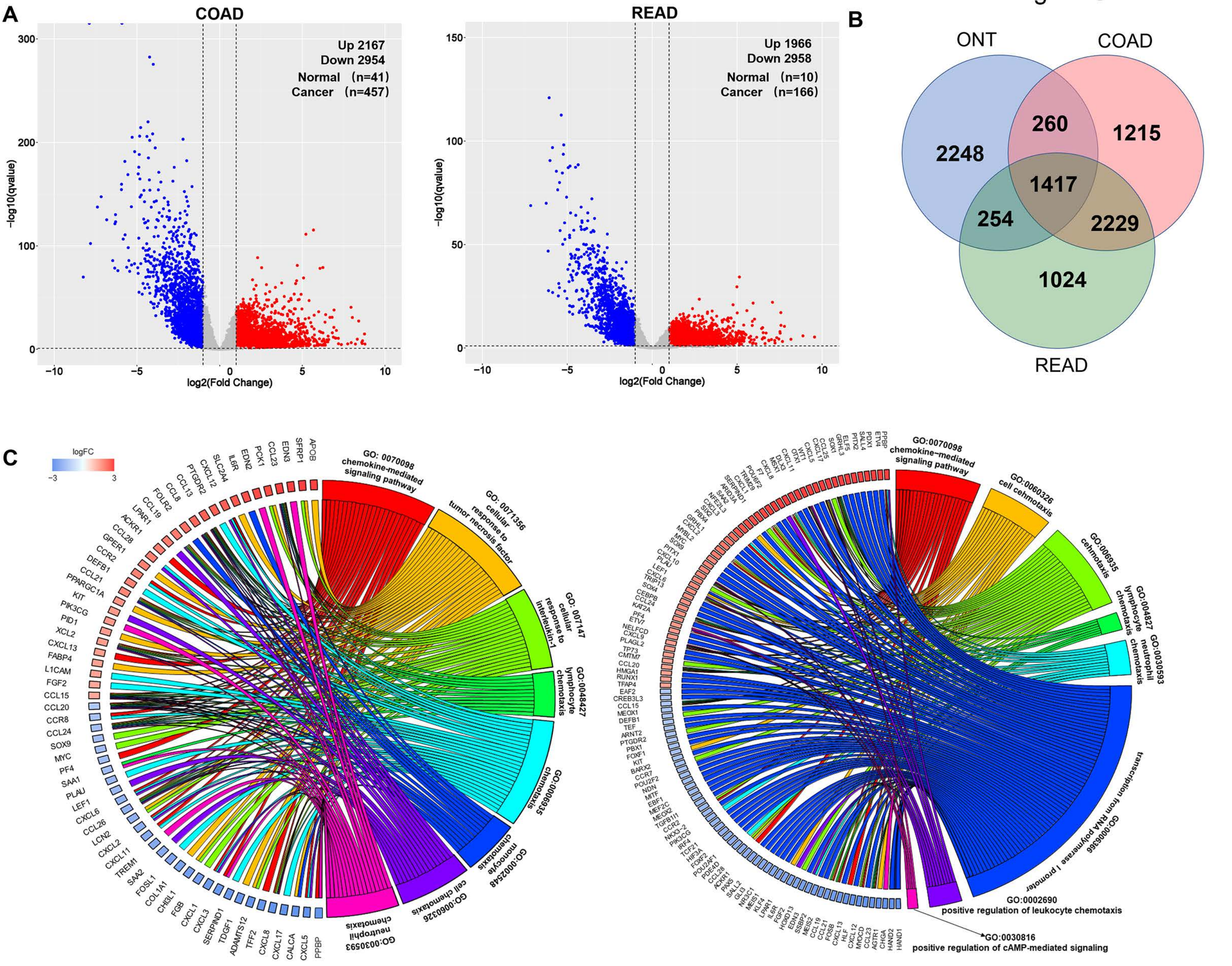
A
COAD
Up 2167
Down 2954
Normal (n=41)
Cancer (n=457)
-log10(qvalue)
log2(Fold Change)
READ
Up 1966
Down 2958
Normal (n=10)
Cancer (n=166)
B
ONT
COAD
READ
2248
260
1215
1417
254
2229
1024
C
logFC
GO: 0070098 chemokine-mediated signaling pathway
GO: 0071356 cellular response to tumor necrosis factor
GO: 007147 cellular response to interleukin-1
GO:0048427 lymphocyte chemotaxis
GO:0006935 chemotaxis
GO:0002548 monocyte chemotaxis
GO:0060326 cell chemotaxis
GO:0030593 neutrophil chemotaxis
GO:0070098 chemokine-mediated signaling pathway
GO:0060326 cell chemotaxis
GO:006935 chemotaxis
GO:004827 lymphocyte chemotaxis
GO:0030593 neutrophil chemotaxis
GO:0006366 transcription from RNA polymerase I promoter
GO:0002690 positive regulation of leukocyte chemotaxis
GO:0030816 positive regulation of cAMP-mediated signaling

Figure S4

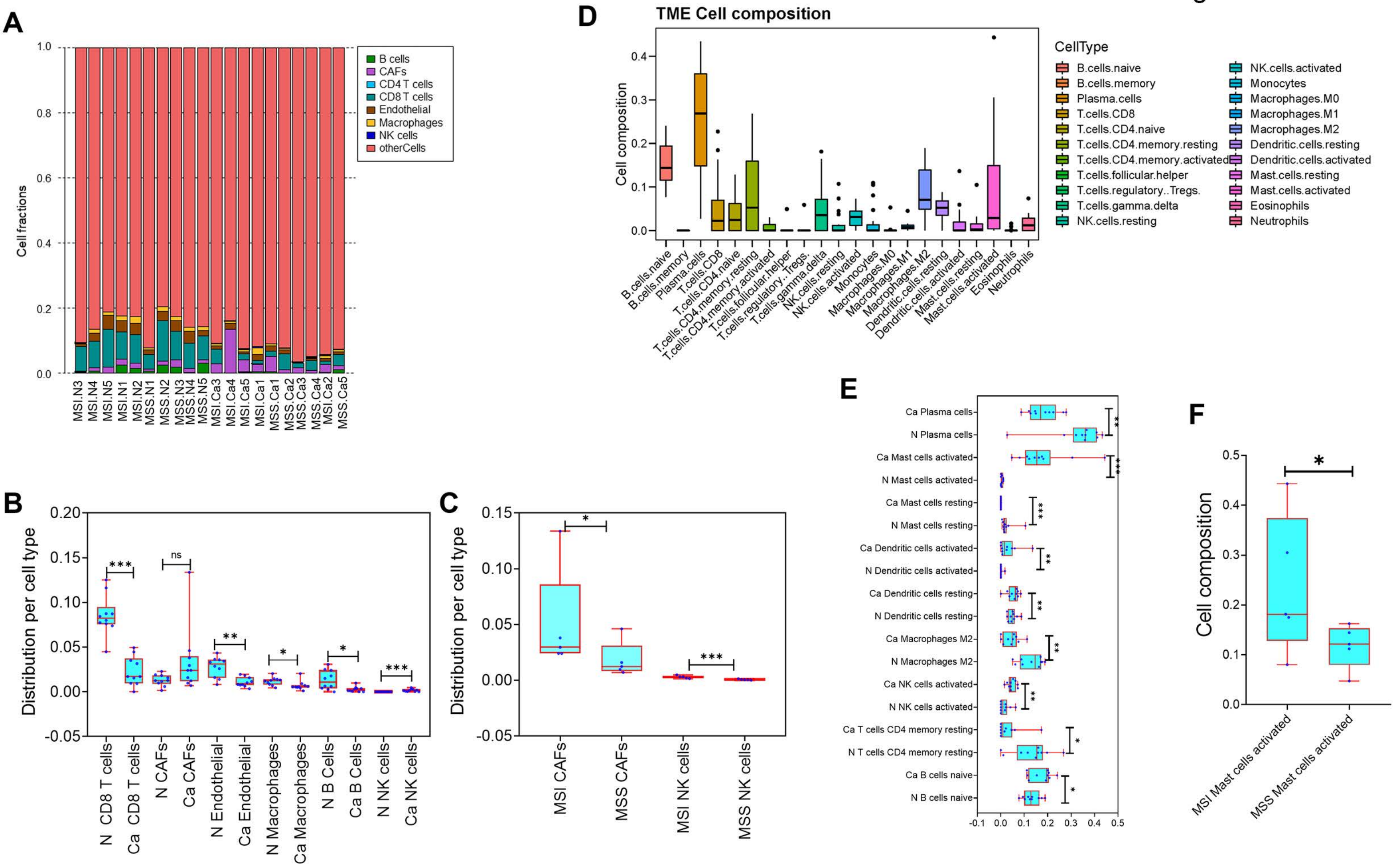
A
Cell fractions
B cells
CAFs
CD4 T cells
CD8 T cells
Endothelial
Macrophages
NK cells
otherCells
MSI.N3
MSI.N4
MSI.N5
MSI.N1
MSI.N2
MSS.N1
MSS.N2
MSS.N3
MSS.N4
MSS.N5
MSI.Ca3
MSI.Ca4
MSI.Ca5
MSI.Ca1
MSS.Ca1
MSS.Ca2
MSS.Ca3
MSS.Ca4
MSI.Ca2
MSS.Ca5
D
TME Cell composition
Cell composition
CellType
B.cells.naive
B.cells.memory
Plasma.cells
T.cells.CD8
T.cells.CD4.naive
T.cells.CD4.memory.resting
T.cells.CD4.memory.activated
T.cells.follicular.helper
T.cells.regulatory..Tregs.
T.cells.gamma.delta
NK.cells.resting
NK.cells.activated
Monocytes
Macrophages.M0
Macrophages.M1
Macrophages.M2
Dendritic.cells.resting
Dendritic.cells.activated
Mast.cells.resting
Mast.cells.activated
Eosinophils
Neutrophils
B
Distribution per cell type
N CD8 T cells
Ca CD8 T cells
N CAFs
Ca CAFs
N Endothelial
Ca Endothelial
N Macrophages
Ca Macrophages
N B Cells
Ca B Cells
N NK cells
Ca NK cells
ns
C
Distribution per cell type
MSI CAFs
MSS CAFs
MSI NK cells
MSS NK cells
E
Ca Plasma cells
N Plasma cells
Ca Mast cells activated
N Mast cells activated
Ca Mast cells resting
N Mast cells resting
Ca Dendritic cells activated
N Dendritic cells activated
Ca Dendritic cells resting
N Dendritic cells resting
Ca Macrophages M2
N Macrophages M2
Ca NK cells activated
N NK cells activated
Ca T cells CD4 memory resting
N T cells CD4 memory resting
Ca B cells naive
N B cells naive
F
Cell composition
MSI Mast cells activated
MSS Mast cells activated

Table S1. The clinical pathological characteristics for 10 subjects using to sequencing

Label	Lymphatic metastasis	Microsatellite	Histopathological grading	Tumor location
MSS-1	-	MSS	middle	right hemicolon
MSS-2	-	MSS	high	right hemicolon
MSS-3	-	MSS	middle	rectal
MSS-4	+	MSS	middle	ascending colon
MSS-5	+	MSS	middle	rectal
MSI-1	-	MSI	middle	right hemicolon
MSI-2	-	MSI	low	right hemicolon
MSI-3	+	MSI	middle	mescolon
MSI-4	+	MSI	ulcerative	right hemicolon
MSI-5	-	MSI	middle	rectal

Table S2. Relationships between clinical pathological characteristic and INHBA levels

characteristic	Total(n=47)	INHBA	*P* value
Sex	M(n=24)	103.5 ± 13.93	0.0023
	F(n=23)	163.7±15.58	
Age	≤65 (=22)	116.3±15.12	0.1505
	>65 (n=25)	145.4±15.38	
CRC	Colon(n=25)	133.0±16.77	0.4852
	Rectal(n=22)	133.0±14.98	
T stage	T1~3 (n=21)	124.8±11.12	0.2634
	T4 (n=26)	139.6±14.25	
N stage	N0~1(n=25)	123.2±14.71	0.2025
	N2(n=22)	144.1±17.26	
M Stage	M0(n=45)	130.2±11.31	0.1610
	M1(n=2)	195.0±75.00	
Tumor size	≤5cm(n=32)	125.6±13.91	0.1634
	>5cm(n=15)	148.7±18.91	
Differentiation	High + moderate (n=35)	134.7±12.96	0.3654
	Low(n=12)	127.9±23.42	
Lymph node status	Negative(n=29)	116.2±12.65	0.0372
	Positive(n=18)	160.0±19.91	
Vascular and nerve invasion	Negative(n=33)	124.2±13.17	0.1000
	Positive(n=14)	153.6±21.08	
